# The Role of the Primary Cilium in Sensing Extracellular pH

**DOI:** 10.3390/cells8070704

**Published:** 2019-07-11

**Authors:** Kimberly F. Atkinson, Rinzhin T. Sherpa, Surya M. Nauli

**Affiliations:** 1 Department of Biomedical & Pharmaceutical Sciences, Chapman University, Irvine, CA 92618, USA; 2 Department of Medicine, Division of Nephrology, University of California Irvine, Irvine, CA 92697, USA

**Keywords:** acidosis, ERK1/2, p38, pH, primary cilia

## Abstract

Biosensors on the membrane of the vascular endothelium are responsible for sensing mechanical and chemical signals in the blood. Transduction of these stimuli into intracellular signaling cascades regulate cellular processes including ion transport, gene expression, cell proliferation, and/or cell death. The primary cilium is a well-known biosensor of shear stress but its role in sensing extracellular pH change has never been examined. As a cellular extension into the immediate microenvironment, the cilium could be a prospective sensor for changes in pH and regulator of acid response in cells. We aim to test our hypothesis that the primary cilium plays the role of an acid sensor in cells using vascular endothelial and embryonic fibroblast cells as in vitro models. We measure changes in cellular pH using pH-sensitive 2′,7′-biscarboxyethy1-5,6-carboxyfluorescein acetoxy-methylester (BCECF) fluorescence and mitogen-activated protein kinase (MAPK) activity to quantify responses to both extracellular pH (pH_o_) and intracellular pH (pH_i_) changes. Our studies show that changes in pH_o_ affect pH_i_ in both wild-type and cilia-less *Tg737* cells and that the kinetics of the pH_i_ response are similar in both cells. Acidic pH_o_ or pH_i_ was observed to change the length of primary cilia in wild-type cells while the cilia in *Tg737* remained absent. Vascular endothelial cells respond to acidic pH through activation of ERK1/2 and p38-mediated signaling pathways. The cilia-less *Tg737* cells exhibit delayed responsiveness to pH_o_ dependent and independent pH_i_ acidification as depicted in the phosphorylation profile of ERK1/2 and p38. Otherwise, intracellular pH homeostatic response to acidic pH_o_ is similar between wild-type and *Tg737* cells, indicating that the primary cilia may not be the sole sensor for physiological pH changes. These endothelial cells respond to pH changes with a predominantly K^+^-dependent pH_i_ recovery mechanism, regardless of ciliary presence or absence.

## 1. Introduction

The normal blood pH level is tightly maintained between 7.35 and 7.45 by the renal and respiratory systems along with buffering mediators in the blood. Lowering of blood pH < 7.35 or acidosis causes several symptoms such as drowsiness, exhaustion, and arrhythmia depending on the type of acidosis. More dramatic changes in pH will induce cytotoxicity and neuronal cell death. The vascular endothelium, which also regulates physical dynamics of blood such as local blood flow and pressure, is best suited to measure local circulating blood pH. Any pH sensor in the body needs to be responsive to extracellular pH fluctuations and regulate downstream mechanisms for homeostatic adaptation. Such adaptation mechanisms include local activation of ion transporters and modulation of channel activity to balance the intracellular ionic gradient, while global regulation is achieved by adjusting the ventilation or renal excretion.

Several studies have shown that mitogen-activated protein kinases (MAPKs) are activated by changes in pH [1,2]. The three known MAPKs, extracellular signal-regulated kinase (ERK1/2 aka p32/p44), p38, and JNK1/2, respond to a variety of environmental stimuli to mediate gene expression, ion transport, cell proliferation, and/or apoptosis [3]. ERK1/2 phosphorylation regulates acid-stimulated vacuolar H^+^-ATPase and Na^+^/H^+^ exchanger (NHE) activation [1,4]. When intracellular pH becomes acidic, ERK1/2 activation acts in parallel with Pyk2 kinase to increase NHE3 activity [5,6]. The MAPK, p38 is activated by various extracellular stress stimuli such as UV light, heat, inflammatory cytokines, and pH changes. Depending on the initial stimuli, substrates of activated p38 include transcription factors and the MAP kinase-activated protein kinase 2 (MK2). MK2 subsequently activates various small heat shock protein 27 (HSP27), lymphocyte-specific protein 1 (LSP1), cAMP response element-binding protein (CREB) among others [7,8].

In the present study, we explore if the primary cilia, distinct from motile cilia in the brain ventricles or Hensen’s node, have a role in acid pH sensation in endothelial cells. Our interest arises from the fact that the primary cilium, a solitary extension of the cell, has been implicated in the sensation of mechanical forces and chemical cues [9,10,11,12]. With the localization of various ion channels, G protein-coupled receptors and receptor-cytoskeletal proteins in the ciliary membrane, the primary cilium is a candidate biosensor that responds to a variety of stimuli [13]. Numerous studies have shown that cilia regulate cytosolic calcium influx and intracellular calcium release upon application of shear stress [12,14,15,16]. The response to blood flow-induced shear stress is very important in the regulation of blood pressure, vascular tone, and vasodilation [13]. As a cellular structure that protrudes out to the vascular lumen and remains in contact with the extracellular milieu, the cilia are poised to be a sensory extension. A recent study on Zebrafish showed localization of acid-sensing ion channels (ASICs), which are proton-gated cation channels, in the cilia of the non-sensory olfactory cell [17]. ASICs are Na^+^ channels activated by external protons and exhibit a rapid response to reduction in extracellular pH (pH_o_) below pH 6.9 [18]. Another Na^+^ channel, the alpha-epithelial sodium channel, has been immunodetected in the cilia and are regulated by flow as well as acidic pH_o_ through Na^+^ gating phenomenon of self-inhibition [19,20,21].

A study by Banizs et al. on cilia-less *Tg737* mice shows that *Tg737* mice have lower intrinsic buffering power when challenged with a weak acid NH_4_^+^ compared to wild-type mice [22]. This indicates that primary cilia might be involved in either sensing pH_o_ change or regulating intracellular pH (pH_i_) in response to pH_o_ changes through ciliary ion transport activity. With the evidence that pH sensitive channels are selectively localized in the cilia of the non-sensory olfactory epithelium [17] and the cilium is known as a sensory organelle of the extracellular milieu [9,12,23,24], we hypothesize that primary cilia could function as pH sensors. We, therefore, examine the role of the primary cilia in acid-activation of MAPK signaling pathways in endothelial cells. We compare the acid response of cilia-less *Tg737* endothelial cells to their wild-type counterparts to examine a possible pH sensing role of the primary cilia.

## 2. Materials and Methods

### 2.1. Cell Culture

Previously isolated and characterized vascular endothelial cells (*Tg737*^+/+^ and *Tg737*^−/−^) were used for the study [23,25]. These cell lines were generated from the same littermates of *Tg737*^+/−^ mice with Balb/C background. The *Tg737* gene encodes for polaris, a structural protein for cilia [26]. These endothelial cells were also immortalized from mice carrying the simian virus-40 (SV40) gene. The promoter of SV40 is regulated by temperature and IFN-γ. As such, cells were grown under permissive conditions in the presence of 0.75 μg/L IFN-γ at 33 °C express SV40 large T antigen regardless of the status of their confluence. The permissive conditions allow cells to hyper-proliferate. When switched to non-permissive conditions in the absence of IFN-γ at 37 °C, the endothelial cells completely shut down the *SV40* gene. Cells under the non-permissive conditions are readily differentiated [23,25].

These cells express common markers for endothelial cells, including eNOS, ICAM-2 (CD102), PECAM-1 (CD31), VE-cadherin (CD144), readily responding to acetylcholine, forming endothelial barrier integrity and having functional intracellular calcium signaling, focal adhesion kinase, calmodulin, Akt/PKB, protein kinase C and eNOS activity [23,25,27]. Aside from abnormal mechanosensory function due to lacking primary cilia, the *Tg737* cilia-less cells also have abnormal cell division [28,29].

Three days prior to experiments, cells were cultured under sterile conditions and maintained at 37 °C in a 5% CO_2_ incubator. Cells were kept in Dulbecco’s Modification of Eagle’s Medium (DMEM), media with 4.5 g/L glucose, l-glutamate, and sodium pyruvate (Corning Cellgro) containing 2% fetal bovine serum (FBS) and 5% penicillin/streptomycin. DMEM with 2% FBS is a low serum condition that promotes ciliation [30]. For NIH3T3 fibroblast cells, growth media consisting of 10% bovine calf serum (BCS) and 5% penicillin/streptomycin in DMEM was used. Cells were grown on poly-l-lysine coated cover glass and incubated with low serum media (2% BCS, 5% penicillin/streptomycin and DMEM) to promote ciliation. To investigate Hedgehog (Hh) signaling in various pH_o_, purmorphamine (Sigma-Aldrich, St. Louis, MO, USA) at a final concentration of 10 μM was used as a positive control. Purmorphamine was added and incubated for 1 h with the cells to induce Hh activation.

### 2.2. Decreased Extracellular pH (pH_o_)

Physiological saline solution (PSS; Table 1) was adjusted to pH_o_ 5.5, 6.0, 6.5, and 7.0 from pH 7.4 (control) using 100 mM HCl. For immunoblot, each 35-mm dish was exposed to media of a given pH for 10 min. Control cells underwent similar treatment with vehicle. Cells were trypsinized and 10^6^ cells transferred into 100 µL 2× Laemelli Sample Buffer (BioRad, Hercules, CA, USA) containing β-mercaptoethanol. Samples were sonicated and heated at 100 °C for 5 min. For tracings of pH_i_ measurement, BCECF-AM-loaded cells were exposed to media of each pH_o_, one at a time for 10 min, sequentially from pH_o_ 7.4 to 5.5. In all our experiments, we maintained our solution osmolality between 290–300 mOsm/L.

### 2.3. Decreased Intracellular pH (pH_i_)

The NH_4_Cl pulse was used to alter pH_i_ using a series of solutions, as previously described [1] and shown in Table 1. All solutions were adjusted to pH_o_ 7.4 and maintained at 37 °C with osmolality between 290–300 mOsm/L. PSS was added to the cells for 5 min, then aspirated. The NH_4_Cl solution (20 mM NH_4_Cl) was then added to cells until intracellular pH stabilized, then aspirated. The cells were immediately incubated in 0K^+^/0Na^+^ solution, causing dissociation of intracellular NH_4_, releasing protons into the cytosol, thus decreasing pH_i_. pH_i_ recovery was accomplished by adding 5K^+^/0Na^+^ solution (5 mM KCl) to the cells and incubation in PSS.

### 2.4. Intracellular pH Measurement

Intracellular pH was measured with 2′,7′-biscarboxyethy1-5,6-carboxyfluorescein acetoxy-methylester (BCECF-AM; Molecular Probes, #B1150, Invitrogen, Eugene, OR, USA). Wild-type and *Tg737* cells were incubated with 5 μM BCECF-AM for 15 min at 37 °C. Images were acquired with a Nikon Eclipse Ti-E inverted microscope using 40× objective and NIS-Elements imaging software (version 4.30, Melville, NY, USA, 2016). Intracellular pH measurements were recorded with emission intensity at wavelength 535 nm. The ratio of emission intensity was determined through excitation wavelengths of pH-dependent 490 nm and an isosbestic point 440 nm. BCECF fluorescence ratio intensity was calibrated to represent intracellular pH using H^+^ ionophore nigericin-containing solutions (Sigma-Aldrich, #N7143). This calibration was performed at the end of each experiment. 20 μM nigericin was used to equilibrate pH_o_ and pH_i_ to pH values of 5.5, 6.0, 6.5, 7.0 and 7.4. Once the 490/440 ratio for each calibration pH value was obtained, the ratio values were fitted to a sigmoidal plot. Subsequent experimental ratios were converted to the pH values.

### 2.5. Immunoblot

All extracellular and intracellular pH manipulations were performed in the same manner for both pH measurement and Western blot analysis. 35 mm dishes were each lysed at different steps of decreased pH_o_ or pH_i_ with NH_4_Cl pre-pulse. Control cells underwent similar treatment with vehicle. Immunoblot of the lysates was used to analyze the phosphorylation of p38 and ERK1/2 in response to decreased pH_o_ or during the different steps of the NH_4_Cl pre-pulse. Blots were probed for β-actin to confirm equal protein loading. Membranes were blocked for 1 h then incubated with primary antibody for 2 h. Primary antibodies include: anti-ERK1/2 (Cell Signaling, #9101), anti-phospho-ERK1/2 (Cell Signaling, #9102), anti-p38 (Abcam, #ab7952), anti-phospho-p38 (Abcam, #ab45381), or anti-β-actin (CellBioLabs, #AKR-002). Cells were rinsed 3× for 10 min then incubated in secondary antibody for 1 h. Secondary antibodies include anti-mouse IgG, HRP-linked (Cell Signaling Technologies, #7076) or anti-rabbit IgG, HRP-linked (Cell Signaling Technologies, #7074). After rinsing three times for 10 min each, membranes were visualized using SuperSignaling West Pico Luminol Enhancers solution (Thermo Scientific, #1859675) and detected with the ChemiDoc from BioRad. Images were acquired and analyzed using ImageLab3.0 software.

### 2.6. Primary Cilia Immunostaining

Cells were grown to confluence on coverslips according to the cell culture conditions mentioned above. The cells were then exposed to media with pH of 5.5, 6.0, 6.5, 7.0 and 7.4 (control) for 5 min. The cells were fixed using 4% paraformaldehyde and 2% sucrose in PBS for 10 min and permeabilized for 5 min in 10% Triton X-100. The cells were incubated with Gli antibody (1:200 dilution in PBS, Abcam, Cambridge, MA, USA) for 16 h at 4 °C, acetylated-α-tubulin (1:10,000 dilution in PBS, Sigma Aldrich, St. Louis, MO, USA) for 1 h at 27 °C followed by fluorescein isothiocyanate (FITC)-conjugated anti-mouse antibody then Texas-red conjugated anti-rabbit antibody (1:1000 dilution in PBS, Vector Labs Burlingame, CA) for 1 h at 27 °C. Slides were mounted with DAPI hard set mounting media (Southern Biotech, Birmingham, AL, USA). Images were acquired using a Nikon Eclipse Ti-E inverted microscope with the NIS-Elements imaging software (version 4.30) in 100× magnification fields with z-stack slices of 0.25 μm. Flat cilia defined by consistent length in four z-slices were measured [31]. The majority of our cilia were flattened on the slide surface, and cilia were measured in three-dimensionally (3D). To ensure cilia flattened on the surface, we included a 3D movie in the supplement (Supplement Movie). We used Nikon NIS-Elements for Advanced Research software to capture and measure all cilia length in 3D. This software package included pre-programmed length analysis through iterations of automatic object recognition followed by image scanning and segmentation, optical flow and 3D object reconstruction. The single-particle tracking was activated only when cilia length was less than 1 μm, especially in *Tg737* cells. In such cases (wild-type and *Tg737* cells), cells with less than 1 μm length of cilia were denoted as non-ciliated cells.

Length measurements of 150 primary cilia was randomly selected using NIS-Elements. To obtain number of cells possessing cilia, six random 100× fields were scanned and a maximum intensity projection created for each field. The total number of cilia and nuclei, as a representation of cell number, was used to calculate ciliated cell percentage. Statistical analysis was performed on Prism GraphPad 8.1.2 software (GraphPad, San Diego, CA, USA).

### 2.7. Scanning Electron Microscopy

Cells were fixed with 2.5% paraformaldehyde/glutaraldehyde in sodium cacodylate buffer for 1 h at 27 °C. Samples were post-fixed with 1% aqueous osmium tetroxide solution. Dehydration was done using ethanol solutions. Samples were further dried with a 2-h incubation in 50% hexamethyldisilazane (HMDS)-ethyl alcohol mixture, followed by two 30-min incubations in 100% HMDS. Micrographs were obtained and analyzed using a Hitachi HD-2300 scanning electron microscope (SEM) [24].

### 2.8. Data Analysis

The rate of pH_i_ changes is denoted as a rate constant of ΔpH_i_ and expressed as dpH/dt (ΔpH_i_ units/min). Because the ΔpH_i_ is defined as rate constant of pH_i_ decreased with respect to time, this pH_i_ was not necessarily decreased at a constant speed. In other words, the changes in pH_i_ could speed up and slow down during the period of measurement. We, therefore, looked at a second order kinetics of these acceleration and deceleration events. The second order kinetics were determined through the tangential rates in the changes of rate constant of ΔpH_i_ using Microsoft Excel software (version 15.32). The mathematical expression to calculate the change of function f at the time t1 is as follows:

$$Changes\;in\;Rate\;Constant\;of\;\Delta pHi=f^{\prime }\left(t1\right)=\;\underset{t2\to t1}{\lim}\frac{f\left(t2\right)-f\left(t1\right)}{t2-t1}$$

All the data shown are mean ± SEM from at least three independent experiments. Data was analyzed using ANOVA test followed by Tukey post-hoc test for multiple groups with *p* < 0.05 being considered as significant. Analysis of data was performed with Prism GraphPad 7 software (GraphPad, San Diego, CA, USA).

## 3. Results

### 3.1. Intracellular Acidosis in Response to Decreasing pH_o_ in Wild-Type and Tg737 Cells

**Figure 1:**
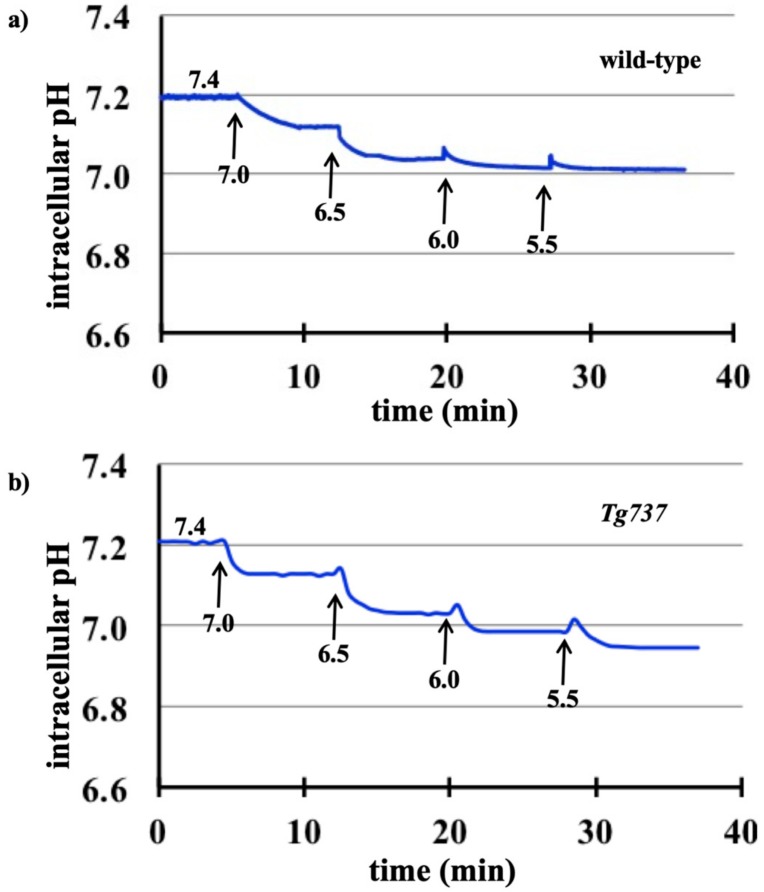

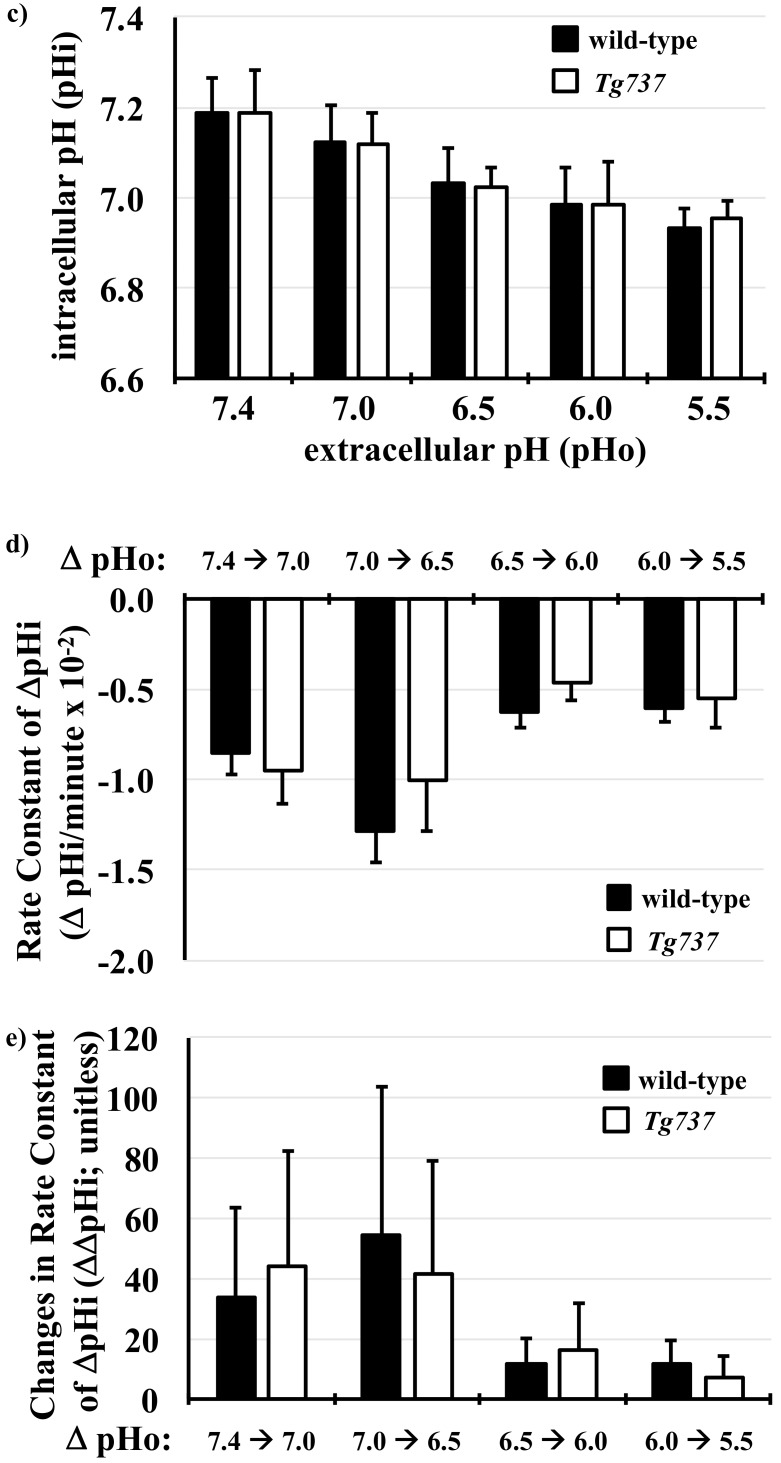
Decreased intracellular pH in response to acidic extracellular pH in wild-type and *Tg737* cells. (**a,b**) Representative tracings of changes in intracellular pH_i_ when wild-type and *Tg737* cells are exposed to media of decreasing extracellular pH (pH_o_) from 7.4 to 5.5. (**c**) As the pH_o_ is decreased, both cell lines show a similar decrease in their pH_i_. (**d**) The rate constant pH_i_ changes (ΔpH_i_) in response step changes in pH_o_ are also similar in both cell lines. A negative value indicates a decrease in pH_i_. (**e**) Changes in ΔpH_i_ (ΔΔpH_i_) are normalized to identify variability within ΔpH_i_ in response to step changes in pH_o_. No variation is observed if there is no acceleration or deceleration (first order kinetic or ΔΔpH_i_ = 0). Highest variation (ΔΔpH_i_ = 100) indicates that an alternate acceleration-deceleration pattern occurred (if acceleration = deceleration, then ΔpH_i_ = 0).

Incubation of cells in PSS of decreasing extracellular pH (pH_o_) from 7.4 to 5.5 acidified the intracellular environment in both wild-type (Figure 1a) and cilia-less *Tg737* cells (Figure 1b). The acidic pH_o_ mediated decrease in pH_i_ was similar in both wild-type and *Tg737* cells (Figure 1c). The rate of pH_i_ changes (ΔpH_i_) was not significantly different between wild-type and *Tg737* cells (Figure 1d). The negative values of ΔpH_i_ indicated that the pH_i_ was decreased in acidified media. There was no significant different between wild-type and *Tg737* cells in the uniformity of or changes in ΔpH_i_ (ΔΔpH_i_; Figure 1e). The positive values of ΔΔpH_i_ indicated that the ΔpH_i_ was predominantly involved in acceleration to decrease pH_i_.

**Figure 2:**
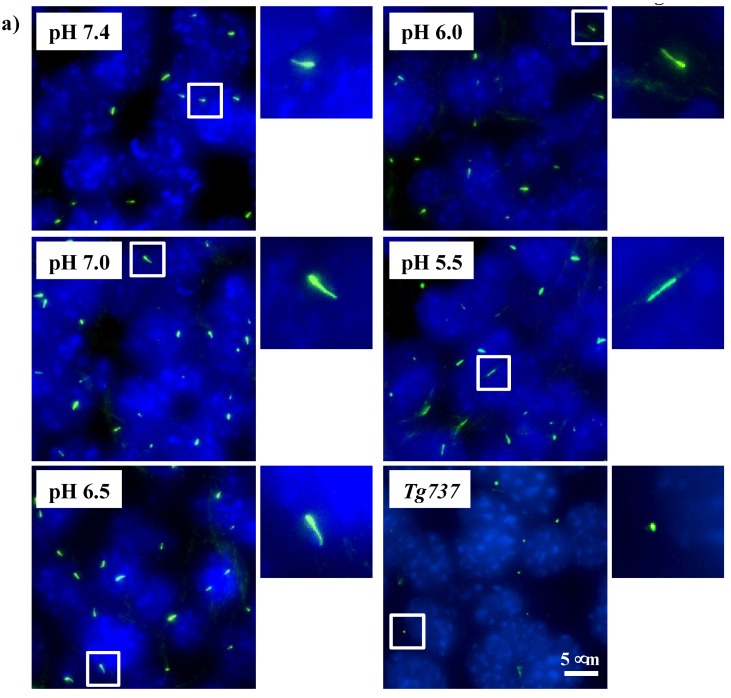

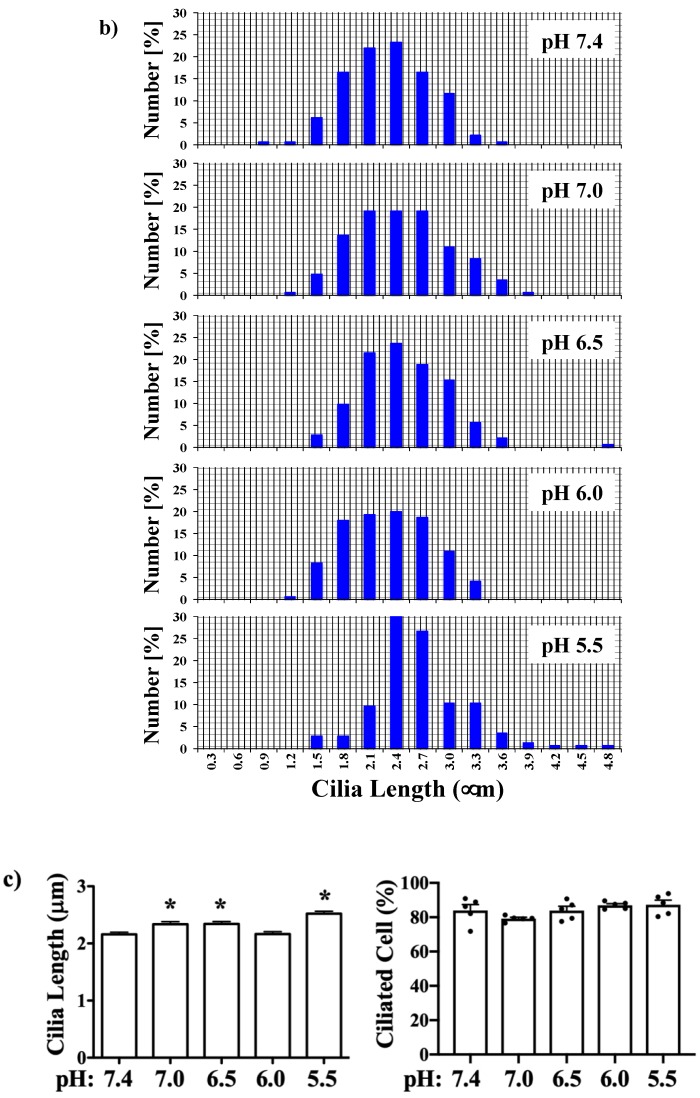
Immunofluorescence staining to study effects of pH_o_ on primary cilia. (**a**) Cells (wild-type and *Tg737*) were stained with ciliary marker (acetylated-α-tubulin; green) and nucleus marker (DAPI; blue). Representative images are shown for wild-type cells at different pH_o_ and *Tg737* at pH_o_ 7.4. White boxes show enlargement of the images to depict the presence of primary cilia. (**b**) The lengths of primary cilia from 50 cells were measured from each preparation (N = 3) and illustrated in the bar graph to depict length distribution within each pH_o_. (**c**) Cilia length was averaged from 150 cells (N = 3; each with 50 randomly selected cells) and the number of cells possessing cilia represented as a percentage with each point representing an individual experimental datapoint. * indicates a significant difference to control pH_o_ 7.4.

To validate that primary cilia remained intact and structurally stable in acidified media, endothelial cilia were examined with ciliary marker acetylated-α-tubulin (Figure 2a). To examine the effect of extracellular pH on the length of primary cilia, the distribution of cilia length as well as ciliation frequency is tabulated in the bar graph (Figure 2b). Compared to control at pH 7.4, a small but significant increase in cilia length was observed at pH_o_ of 7.0, 6.5 and 5.5 while there were no significant differences observed in ciliation frequency (Figure 2c). There were no apparent differences in the cilia formation at different extracellular pH levels. Approximately 80–85% of wild-type cells were ciliated in acidified media, as well as at pH 7.4. For cilia-less *Tg737* cells, the only representative image at pH 7.4 is shown with no further apparent differences in acidified media. There were no cilia length increase in *Tg737* cells at various pH_i_.

Most importantly, the data shows that wild-type cells continued to possess primary cilia without any structural aberrations in an acidic environment. Further validation with another ciliated cell line, NIH3T3, was conducted to observe changes in ciliary length or ciliation frequency when challenged with acidified media (Supplement Figure S1–S3). Lower acidic media was able to significantly increase ciliary length at pH_o_ of 6.5 and 5.5 while ciliation frequency remained unchanged at lower pH_o_. Among the many signaling activities related to the primary cilia, Hedgehog signaling (Hh) is unique in translocating activated receptors and proteins to the cilia [32]. To observe changes in functional role of the primary cilia under our experimental conditions, we used purmophamine to activate the Hh pathway. After observing no effect of Hh activation on ciliary length or ciliation frequency of the cells, we studied if acidified media induced Hh signaling, as shown by Gli translocation to the ciliary tip in NIH3T3 cells (Supplement Figure S3). There is also no apparent structural defect in cilia following a decrease in intracellular pH (Supplement Figure S4).

### 3.2. MAPK Activation in Response to Decreasing pH_o_ in Wild-Type and Tg737 Cells

**Figure 3:**
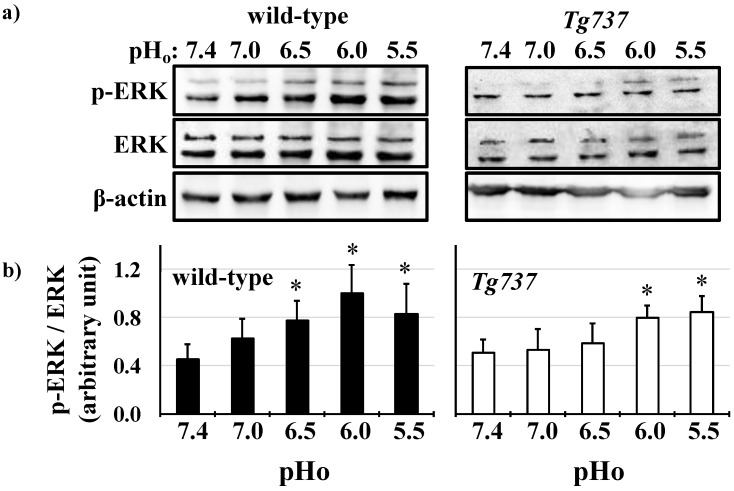
Decreasing extracellular pH increases ERK1/2 phosphorylation in wild-type and *Tg737* cells. (**a**) Representative immunoblots show ERK1/2 phosphorylation in wild-type and *Tg737* cells as they are exposed to media of decreasing extracellular pH (pH_o_) from 7.4 to 5.5. (**b**) Bar graph shows the mean p-ERK/ERK, where wild-type cells phosphorylate ERK1/2 at pH of 6.5 while the same level of phosphorylation occurs at a lower pH of 6.0. * indicates *p* < 0.05 as compared to control pH_o_ 7.4; N = 10 for wild-type cells and N = 10 for *Tg737* cells.

**Figure 4:**
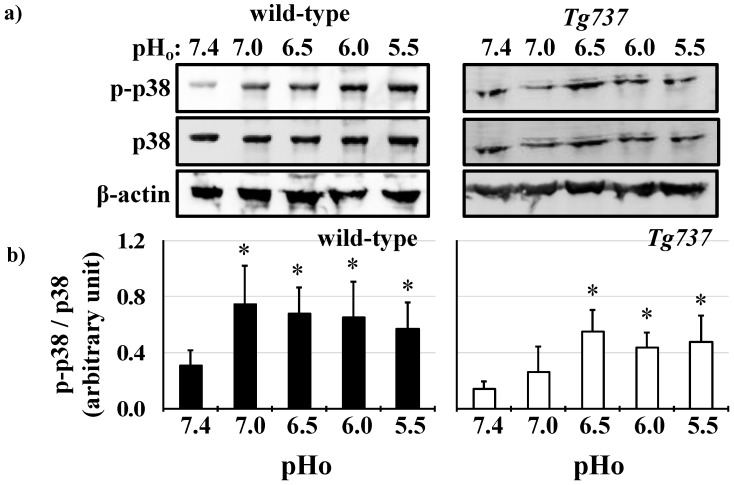
p38 phosphorylation by decreased extracellular pH in wild-type and *Tg737* cells. (**a**) Representative immunoblots show p38 phosphorylation in wild-type and *Tg737* cells as they are exposed to media of decreasing extracellular pH (pH_o_) from 7.4 to 5.5. (**b**) Bar graph shows mean p-p38/p38. Significant increase in p-38 phosphorylation occurs when pH changes from 74. to 7.0. But in *Tg737* cells the pH had to drop to 6.5 before significant changes in p38 phosphorylation was observed. * indicates *p* < 0.05 as compared to control pH_o_ 7.4; N = 8 for wild-type cells and N = 8 for *Tg737* cells.

Both wild-type and *Tg737* cells exhibited MAPK phosphorylation in response to decreased pH_o_. Significant increase of ERK1/2 phosphorylation occurred at pH_o_ 6.5 in wild-type cells (178% increase versus control pH_o_ 7.4) and persisted at pH_o_ 6.0 and pH_o_ 5.5 (227% and 189% increase in ERK1/2 phosphorylation, respectively versus control pH_o_ 7.4) (Figure 3a). ERK1/2 phosphorylation occurred at pH_o_ 6.0 in *Tg737* cells (140% increase versus control pH_o_ 7.4) (Figure 3b). Another MAPK, p38 was phosphorylated at pH_o_ 7.0 in wild-type cells (242% increase versus control pH_o_ 7.4) and persisted at pH_o_ 6.0 and pHo 5.5 (227% and 189% increase in p38 phosphorylation, respectively versus control pH_o_ 7.4) (Figure 4a). p38 phosphorylation occurred at pH_o_ 6.5 in *Tg737* cells (393% increase versus control pH_o_ 7.4) and persisted through pH_o_ 5.5 (321% and 360% increase in p38 phosphorylation at pH_o_ 6.0 and 5.5, respectively, versus control pH_o_ 7.4) (Figure 4b). *Tg737* cells required higher acidic conditions to increase MAPK phosphorylation than the wild-type cells. There did not seem to be a significant impairment in cilia-less *Tg737* cells’ ability to sense and respond to decreased pH_o_, but we find a lower pH threshold for activation of MAPK in *Tg737* compared to wild-type cells.

### 3.3. NH_4_Cl Pre-Pulse Induces Intracellular Acidosis in Wild-Type and Tg737 Cells

**Figure 5:**
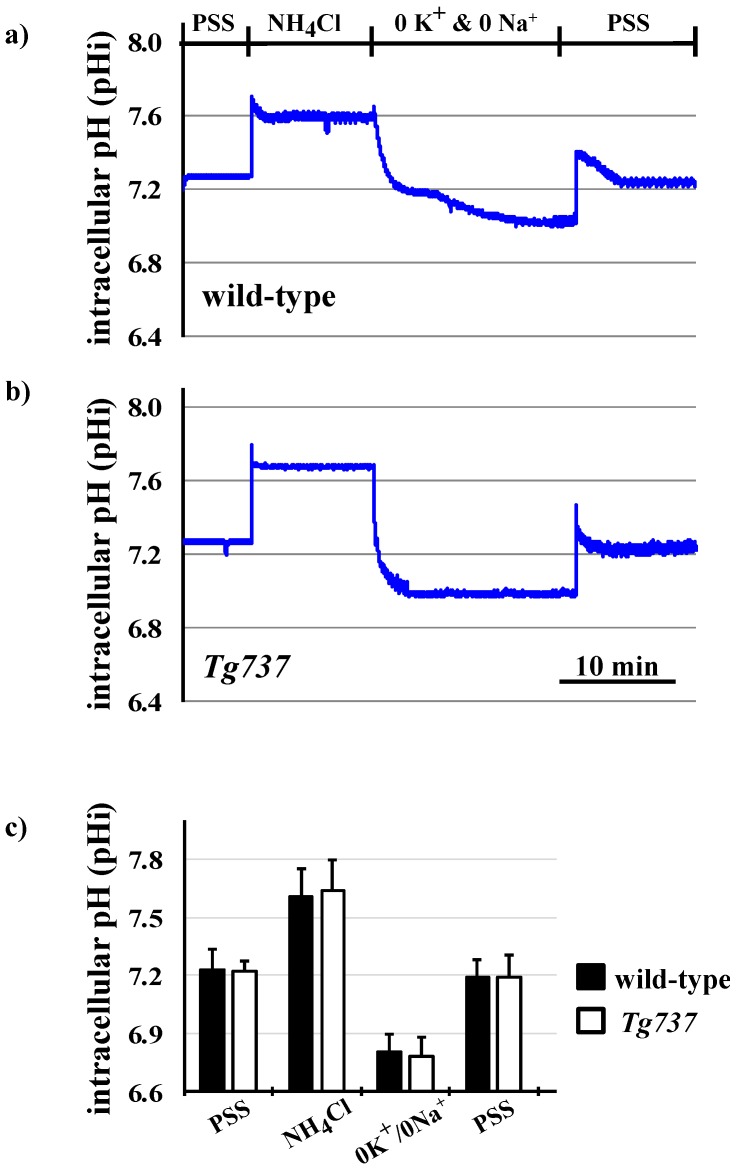
Changes in pH_i_ during the NH_4_Cl pre-pulse in wild-type and *Tg737* cells. (**a,b**) Representative tracings of changes in intracellular pH when wild-type and *Tg737* cells are exposed to solutions of the NH_4_Cl pre-pulse in Na^+^- and K^+^-devoid solution for pH_i_ recovery. (**c**) Bar graph shows the summary of mean changes in pH_i_ recovery in wild-type and *Tg737* cells. N = 5 for wild-type cells and N = 3 for *Tg737* cells.

To eliminate the effect of pH_o_ and observe intracellularly restricted pH acidosis NH_4_Cl pre-pulse was used to lower pH_i_. Changes in pH_i_ during the NH_4_Cl pre-pulse using Na^+^- and K^+^-containing PSS for pH_i_ recovery are depicted in representative tracings (Figure 5a,b of wild-type and *Tg737* cells, respectively) and data is summarized in Figure 5c. In wild-type cells, NH_4_Cl caused an increase in intracellular pH (0.014 ± 0.002 pH units/min); in *Tg737* cells, NH_4_Cl also increased the pH_i_ (0.019 ± 0.003 pH units/min). In the absence of sodium and potassium, the pH_i_ decreased in both cell types (0.028 ± 0.004 pH units/min in wild-type cells and 0.020 ± 0.003 pH units/min in *Tg737* cells, from NH_4_Cl conditions). pH_i_ recovery occurred upon addition of the Na^+^- and K^+^-containing PSS solution, at a rate of 0.006 ± 0.0001 pH units/min in wild-type cells, and more significantly in *Tg737* cells at a rate of 0.031 ± 0.003 pH units/min, from 0K^+^/0Na^+^ conditions. Because rates did not increase during incubation in the 0K^+^/0Na^+^ solution, Na^+^- or K^+^-dependent transport is likely responsible for endothelial cells’ recovery from decreased pH_i_. Another inference is that Na^+^- and K^+^-independent transport may not be present or activated by intracellular acidosis, in vascular endothelial cells.

To validate that primary cilia remained intact and structurally stable after intracellular acidification independent of pH_o_, endothelial cilia were examined with ciliary marker acetylated-α-tubulin (Supplement Figure S5). To examine the effect of pH_i_ acidification on the length of primary cilia, the distribution of cilia length as well as ciliation frequency is tabulated in the bar graph (Supplement Figure S6). Compared to control at pH_i_ 7.4, a small but significant decrease in cilia length was observed at pH_i_ of 7.0 while there were no significant differences observed in ciliation frequency. There were no apparent differences in the cilia formation between pH_i_ 7.4 and 7.0. Approximately 80–85% of wild-type cells were ciliated. There were no cilia length recovery in *Tg737* cells at pH_i_ of 7.0.

### 3.4. Intracellular Acidosis Activates MAPK Signaling Pathways

**Figure 6:**
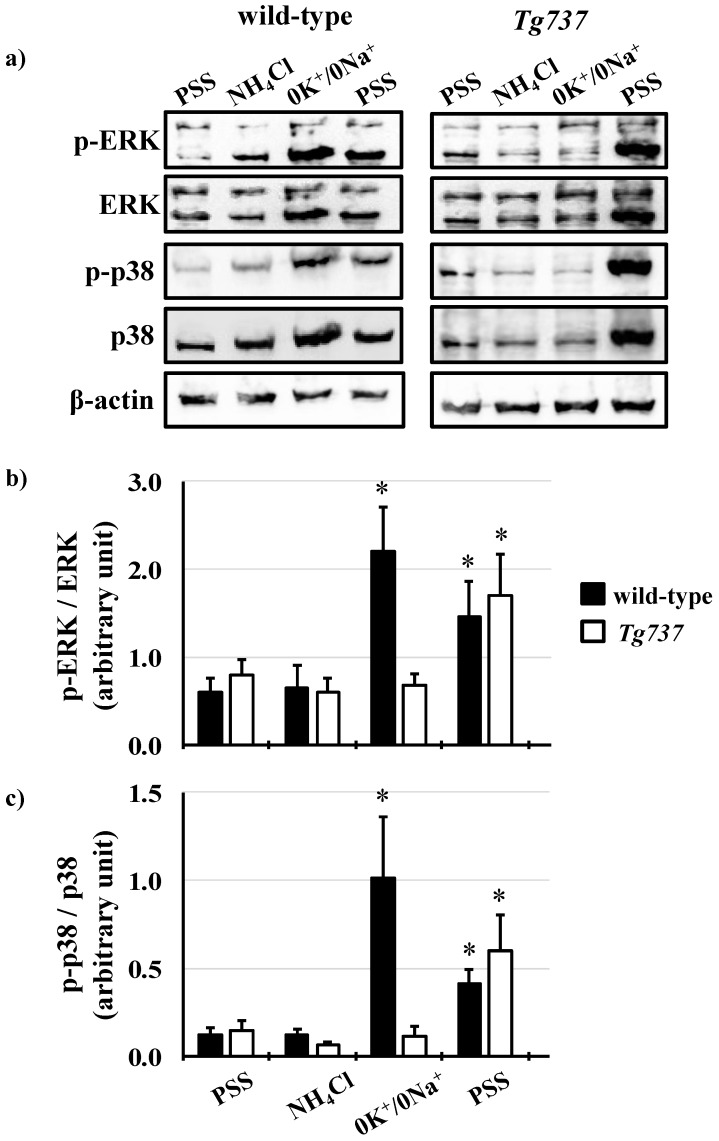
ERK1/2 and p38 phosphorylation during the NH_4_Cl pre-pulse in wild-type and *Tg737* cells. (**a**) Representative immunoblots show ERK1/2 and p38 phosphorylation in wild-type and *Tg737* cells as they are exposed to different solutions during the NH_4_Cl pre-pulse. (**b**,**c**) Bar graphs show mean p-ERK/ERK and p-p38/p38. * indicates *p* < 0.05 as compared to control conditions; for ERK1/2 N = 6 for wild-type cells and N = 5 for *Tg737* cells; for p38 N = 5 for wild-type cells and N = 5 for *Tg737* cells.

MAPK phosphorylation during the NH_4_Cl pre-pulse was similar in both cell types, with slight variations (Figure 6a). ERK1/2 phosphorylation at acid pH_i_ during the 0K^+^/0Na^+^ step of the NH_4_Cl pre-pulse occurred in only wild-type cells (3.7-fold increase in ERK1/2 phosphorylation versus control conditions) (Figure 6b). ERK1/2 phosphorylation was observed during pH_i_ recovery in both wild-type and *Tg737* cells (2.1-fold and 2.3-fold respective increases in phosphorylation versus control conditions). p38 phosphorylation pattern varied between the wild type and cilia-less *Tg737* as well. In wild-type cells, p38 phosphorylation occurred at low pH_i_ during the 0K^+^/0Na^+^ step of the NH_4_Cl pre-pulse (10-fold increase in phosphorylation versus control conditions); in *Tg737* cells, p38 phosphorylation was dampened, occurring only during pH_i_ recovery (4.0-fold increase in p38 phosphorylation versus control conditions) (Figure 6c).

### 3.5. Effects of K^+^ on pH_i_ Recovery and on MAPK Phosphorylation during the NH_4_Cl Pre-Pulse in Wild-Type and Tg737 Cells

**Figure 7:**
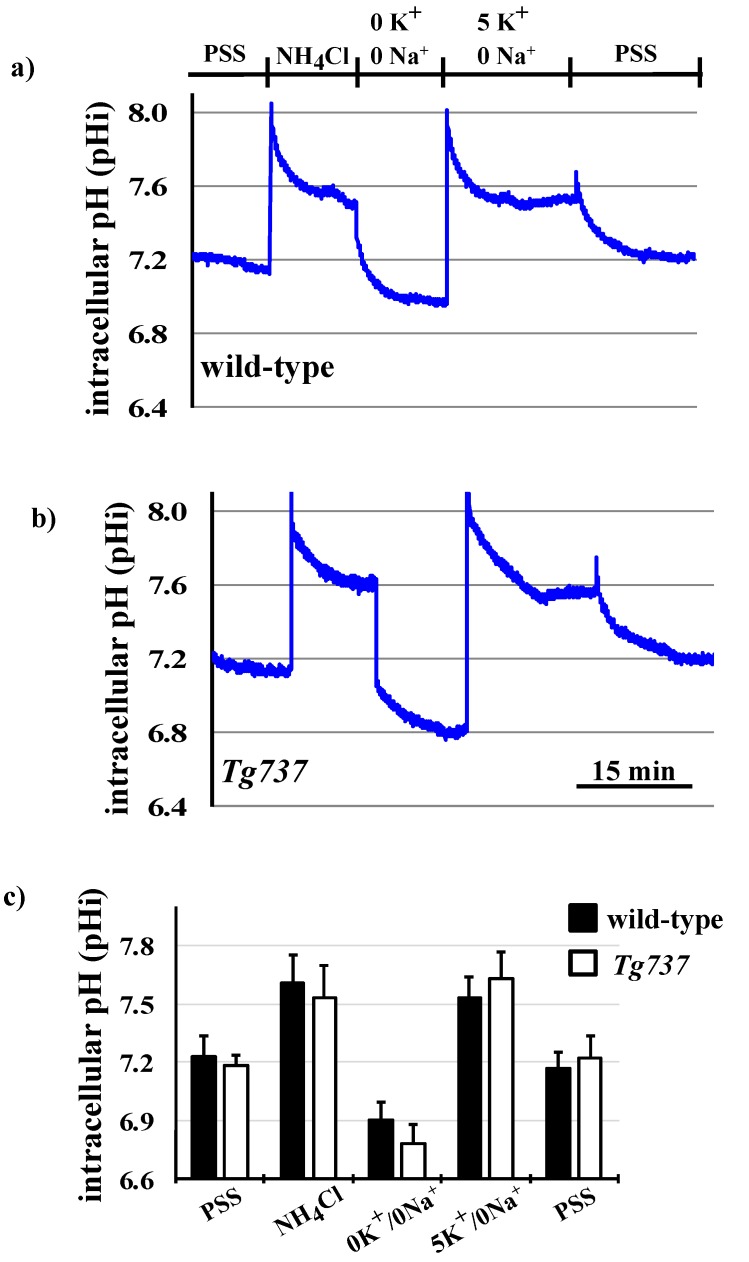
Changes in pH_i_ during the NH_4_Cl pre-pulse in wild-type and *Tg737* cells. (**a,b**) Representative tracings of changes in intracellular pH when wild-type and *Tg737* cells are exposed to solutions of the NH_4_Cl pre-pulse including the 5 mM K^+^ solution during pH_i_ recovery of wild-type and *Tg737* cells. (**c**) Bar graph shows the summary of pH_i_ recovery data from pre-pulse tracings in wild-type and *Tg737* cells. N = 5 for wild-type cells and N = 5 for *Tg737* cells with the 5K^+^/0Na^+^ solution.

Potassium-containing 5K^+^/0Na^+^ solution could be used to study K^+^-dependent pH_i_ recovery [1]. When the NH_4_Cl pre-pulse was expanded to include potassium-containing 5K^+^/0Na^+^ solution in between the 0K^+^/0Na^+^ and PSS solutions during pH_i_ recovery, the pH_i_ increased very rapidly in wild-type cells and *Tg737* cells, highlighting the importance of K^+^-dependent transporter activity during pH_i_ recovery in vascular endothelial cells, shown in representative tracings in Figure 7a,b, and summarized in Figure 7c. Addition of potassium-containing 5K^+^/0Na^+^ solution drove the pH_i_ up in both cell types (0.030 ± 0.003 pH units/min in wild-type cells and 0.035 ± 0.003 pH units in *Tg737* cells, versus 0K^+^/0Na^+^ conditions). Interestingly, addition of PSS at the end of the pulse returned the pH_i_ to a normal level by driving the pH_i_ down at a rate of 0.036 ± 0.005 pH units/min in wild-type cells and 0.035 ± 0.006 pH units/min in *Tg737* cells, from 5K^+^/0Na^+^ conditions.

**Figure 8:**
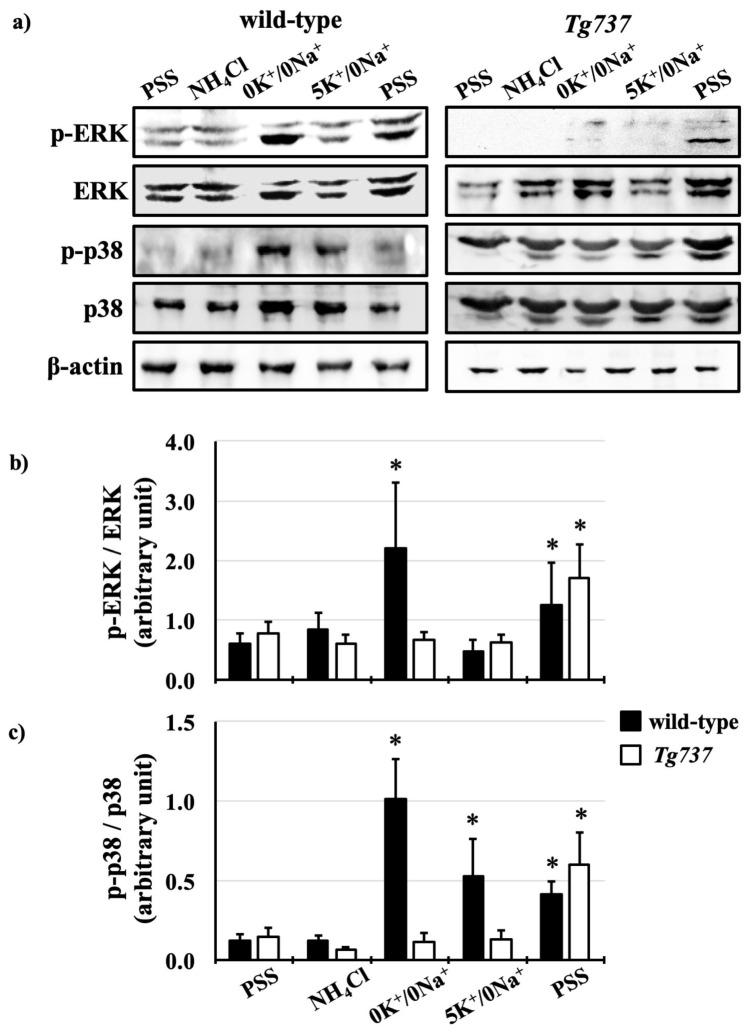
Effect of K^+^ on ERK1/2 and p38 phosphorylation during the NH_4_Cl pre-pulse in wild-type and *Tg737* cells. (**a**) Representative immunoblots show ERK1/2 and p38 phosphorylation in wild-type and *Tg737* cells as they are exposed to different solutions during the NH_4_Cl pre-pulse. (**b,c**) Bar graphs show mean p-ERK/ERK and p-p38/p38, respectively. * denotes *p* < 0.05 as compared to control conditions. For ERK1/2, N = 8 for wild-type cells and N = 8 for *Tg737* cells; for p38, N = 7 for wild-type cells and N = 5 for *Tg737* cells.

Addition of potassium-containing 5K^+^/0Na^+^ solution during pH_i_ recovery also influenced MAPK phosphorylation, more specifically p38 phosphorylation (Figure 8). In wild-type cells, p38 phosphorylation coincided with ERK1/2 phosphorylation at acidified pH_i_, during the 0K^+^/0Na^+^ step of the NH_4_Cl pre-pulse (10-fold increase in phosphorylation versus control conditions), and p38 phosphorylation persisted through pH_i_ recovery using the 5K^+^/0Na^+^ solution (5.5-fold increase in phosphorylation versus control conditions). In *Tg737* cells, p38 phosphorylation occurred later during pH_i_ recovery with the PSS solution (4.0-fold increase in phosphorylation versus control conditions). Both ERK1/2 and p38 phosphorylation were absent in *Tg737* cells as compared to wild-type cells, where significant increases in MAPK phosphorylation occurred after inducing intracellular acidosis.

## 4. Discussion

In the present studies, we assessed the role of primary cilia in pH sensing of vascular endothelial cells. We compared intracellular responses in wild-type and cilia-less *Tg737* mutant cells against extracellular pH changes and obtained three main results. First, intracellular pH homeostasis was not significantly different between the wild-type and *Tg737* cells. Second, phosphorylation of two mitogen-activated kinases, p38 and ERK1/2, were increased by lowering extracellular pH in both the wild-type and the *Tg737* cells, but for *Tg737* cells the same extent of phosphorylation needed a stronger acidic condition. Third, when the cells were exposed to 0K^+^/0Na^+^ and 5K^+^/0Na^+^ solutions after the NH_4_Cl solution, the phosphorylation of p38 and ERK1/2 was enhanced only in the wild-type cells. 

Wild-type and *Tg737* cells have similar responses to decreased extracellular pH (pH_o_), including decreased intracellular pH (pH_i_) relative to drops in pH_o_ and acute ERK1/2 and p38 phosphorylation at pH_o_ < 6.0 (Figure 1, Figure 2, Figure 3 and Figure 4). Diminished MAPK phosphorylation was observed in *Tg737* cells compared to wild-type cells at certain pH_o_. A significant finding from these experiments was that MAPK phosphorylation in vascular endothelial cells when exposed to low pH_o_ may be associated to the cilia or there could be inherited machinery differences between wild-type and *Tg737* cells in terms of ERK1/2 and p38 phosphorylation.

An acidic environment increased the length of primary cilia in wild-type cells, whereas isolated acidification of intracellular pH decreased cilia length. The *Tg737* cells remained cilia-less under all conditions. Similar to endothelial cells, the NIH3T3 fibroblast cells also presented with longer cilia when challenged with an acidic environment. The physiological significance of this cilia length changes is not clear at present. However, it has been speculated that cilia length could be used as a cellular marker in response to injury or environmental insults [33,34,35]. SEM micrographs showed no structural defects in the cilia after exposure to acidified media. With the aim of finding any functional effects of acidified media in the cilium, we looked at possible acidic pH_o_ induced Hh signaling but found no unexpected activation of Hh.

The next set of experiments were designed to bypass extracellular pH sensing by lowering intracellular pH only (Figure 5, Figure 6, Figure 7 and Figure 8). In terms of pH_i_ acidification, NH_4_Cl pre-pulse procedure lowered control and *Tg737* cell pH_i_ in the same manner. *Tg737* cells showed no ERK1/2 or p38 phosphorylation in response to decreased intracellular pH, compared to wild-type cells, consistent with a lower pH requirement as seen in pH_o_ induced intracellular acidification. Phosphorylation of p38 during K^+^-mediated pH_i_ recovery is absent in *Tg737* cells but this does not produce any differences in pH_i_ recovery pattern in comparison to wild-type cells. From our findings we conclude that endothelial cilia are unlikely to serve as the only acid sensing organelle but may be involved in buffering capacity of cytosolic pH in vascular endothelial cells. The significance in this could mean that ciliopathy (abnormal cilia) may have very little direct role in the physiological acid-base imbalance.

Acid-induced MAPK activation has been observed in the renal epithelial cells [36]. Our studies show that acid activation of MAPK, p38, is relevant in endothelial cells and may be involved in the regulation of acid-mediated transport. Consistent with our observation, Flacke et al. showed that Wistar rat coronary endothelial cells exposed to acidosis (pH 6.4) led to a transient activation of p38 and Akt kinases, which are essential for protection against apoptosis [37].

The importance of K^+^ channels in pH_i_ recovery in vascular endothelial cells has been highlighted in this study, with profound increases in intracellular pH upon addition of K^+^-containing solution following intracellular acidosis. Our data on K^+^-dependent pH_i_ recovery indicates that K^+^-transporters are primarily activated by low pH_i_, which could include K^+^-channels, Na^+^/K^+^ pumps, and/or Na^+^/K^+^/2Cl^−^ cotransporters. Studies have shown that ATP-sensitive K^+^ channels are activated directly by intracellular but not by extracellular acidosis. This has been established in rat basilar artery where pH_i_-acidification mediated dilation was blocked by glibenclamide, an inhibitor of ATP-sensitive potassium channels [38]. Future studies will be needed to determine the precise potassium channel, pump, or transporter responsible for K^+^-dependent pH_i_ recovery in vascular endothelial cells.

A single, universal pH/acid sensor in the cardiovascular and renal systems that regulates MAPK pathways and ion transport has yet to be identified, but there are several possible candidates. These putative pH sensors expressed by vascular endothelial cells will lie upstream of acid-activated ERK1/2 and p38, and may include epidermal growth factor receptor (EGFR) [4], an acid-sensing ion channel (ASIC) [39], or a G-protein coupled receptor. The GPCR GPR4 is known to be acid-activated [40,41] and regulates potassium-driven transport to maintain pH [42]. GPR4-null mice have minor defects in renal acid excretion and mild metabolic acidosis. GPR4 deficiency also affects the quality of small blood vessels during angiogenesis [43]. In vascular endothelial cells, acidosis activation of GPR4 stimulates inflammatory responses [43]. With p38 being the notorious inflammatory MAPK [3] and its activation being clear in response to acid pH, GPR4 would be a promising pH sensor in vascular endothelial cells in the regulation of p38-mediated signaling pathways described here.

Other good candidates for a pH sensor in vascular endothelial cells are acid-sensing ion channels (ASICs), which are ligand-gated and amiloride-sensitive cation channels activated by extracellular H^+^ [44]. ASICs are members of the degenerin/epithelial sodium channel (DEG/ENaC) superfamily and contain an acidic pocket responsible for acid-dependent gating of sodium and calcium, albeit to a lesser degree. ASICs are expressed in the central and peripheral nervous system, including afferent tissues such as skin, cardiovascular system, muscle, joint, teeth, vestibular, and visceral cells [45].

With the primary cilium being established as a sensor of different mechanical and biochemical cues, we test whether the primary cilium has a role in sensing and transducing pH changes. We compare acidosis response using vascular endothelial cells as an in vitro model compared to cilia-less *Tg737* cells. Our study on non-motile primary cilia may not be extrapolated to the motile cilia in developing nodes or in the brain ventricles. Nonetheless, our data shows that acid-activation of p38- and ERK1/2-mediated signaling pathways regulate ion transport to maintain acid-base homeostasis in endothelia. We showed that pH_i_ recovery after an NH_4_Cl pre-pulse in vascular endothelial cells is predominantly a K^+^-dependent process. We also observed that a more acidic pH_o_ was needed to induce MAPK phosphorylation in cilia-less *Tg737* cells compared to wild-type cell. NH_4_Cl pre-pulse is a technique used to create pH_o_-independent pH_i_ acidification, in this scenario pH_i_ recovery was seen to be delayed in *Tg737* cells. Therefore, we conclude that the primary cilium, a known cardiovascular mechanosensor [23,46,47] and chemoreceptor [24,48,49] is not the sole sensor for acid sensation but does influence the pH threshold for MAPK kinase phosphorylation. Future studies to examine the identity of the acid sensors distributed in the cilia might be able to detail the nuanced role of primary cilia or *Tg737* deletion that might affect the buffering capacity of the *Tg737* mice model.

## Figures and Tables

**Table 1:** Composition of solutions used in the NH_4_Cl pre-pulse. Solution names are listed along the first row, and the composition of each is shown in each column. Components of each solution are in mM. HEPES = (4-(2-hydroxyethyl)-1-piperazineethanesulfonic acid); NMDG = N-methyl-D-glucamine.

Solution	PSS	NH_4_Cl	0K^+^/0Na^+^	5K^+^/0Na^+^
CaCl_2_	1.8	1.8	1.8	1.8
MgSO_4_	0.8	0.8	0.8	0.8
Glucose	5.5	5.5	5.5	5.5
HEPES	10	10	10	10
NaCl	135	0	0	0
KCl	5	0	0	5
NH_4_Cl	0	20	0	0
NMDG	0	120	140	135

## Supplementary Materials

The following are available online at https://www.mdpi.com/2073-4409/8/7/704/s1, Supplement Movie: 3D reconstruction of z-stack (0.25 μm slices) from NIH3T3 cells highlighting the cilia (green), Gli (red) and nucleus (blue).Figure S1: NIH3T3 were stained with ciliary marker (acetylated-α-tubulin; green), Gli (red) and nucleus marker (DAPI; blue). Figure S2: The lengths of primary cilia from 150 NIH3T3 cells were measured from each preparation (N = 3; each 50 randomly selected cilia). Figure S3: (a) Cilium length of NIH3T3 cells before and after Hh activation or different acidic pH_o_ exposures was averaged. (b) The percentage of cells with cilia is shown. (c) The percentage of cells is shown with Gli localization to the cilia. Figure S4: Electron micrographs of endothelial cells at pH 7.4 (top) and pH 5.5 (bottom). Figure S5: Endothelial cells were stained with ciliary marker (acetylated-α-tubulin; green) and nucleus marker (DAPI; blue). Figure S6: The lengths of primary cilia from 150 endothelial cells were measured from each preparation before and after NH_4_Cl pre-pulse in 0 Na^+^/K^+^ solution (N = 3; each 50 randomly selected cilia).
