# Supplementary material for: The Role of the Primary Cilium in Sensing Extracellular pH
Source: Cells. 2019 Jul 11;8(7):704. doi: 10.3390/cells8070704 (PMC6679169; doi:10.3390/cells8070704)
Supplement: Supplementary file 1 [file cells-08-00704-s001.zip › cells-515832 supplementary 7.11/SuppInfo.pdf]

The role of the primary cilium in sensing extracellular pH

Kimberly F. Atkinson[1,#], Rinzhin T. Sherpa[1,#], Surya M. Nauli[1,2,*]

[1] Department of Biomedical & Pharmaceutical Sciences, Chapman University, Irvine, CA; kfisher@chapman.edu (K.F.A); sherp101@mail.chapman.edu (R.T.S)

[2] Department of Medicine, Division of Nephrology, University of California Irvine, Irvine, CA

[#] These authors contributed equally to the work

[*] Correspondence: nauli@chapman.edu; snauli@uci.edu; Tel.: 714-516-5480

Supplemental Information:

1. **Supplement Movie:** 3D reconstruction of z-stack (0.25 µm slices) from NIH3T3 cells highlighting the cilia (green), Gli (red) and nucleus (blue).

2. **Supplement Figures:** 1-6

Supplement Figure 1

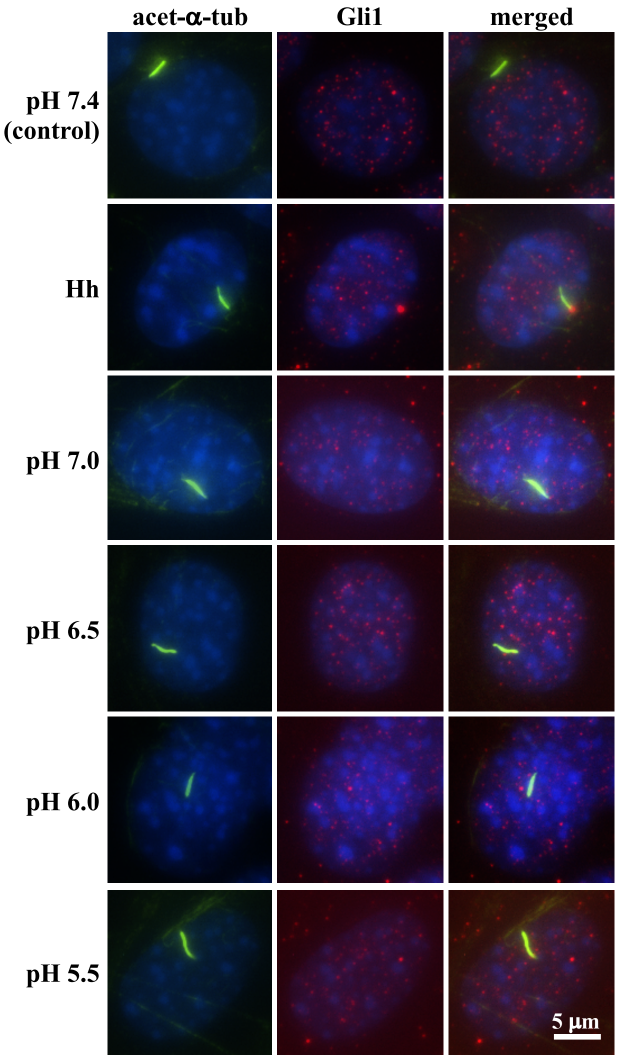

Supplement Figure 1. NIH3T3 were stained with ciliary marker (acetylated-α-tubulin; green), Gli (red) and nucleus marker (DAPI; blue). Representative images are shown at different pH_o or with Hh activation.

Supplement Figure 2

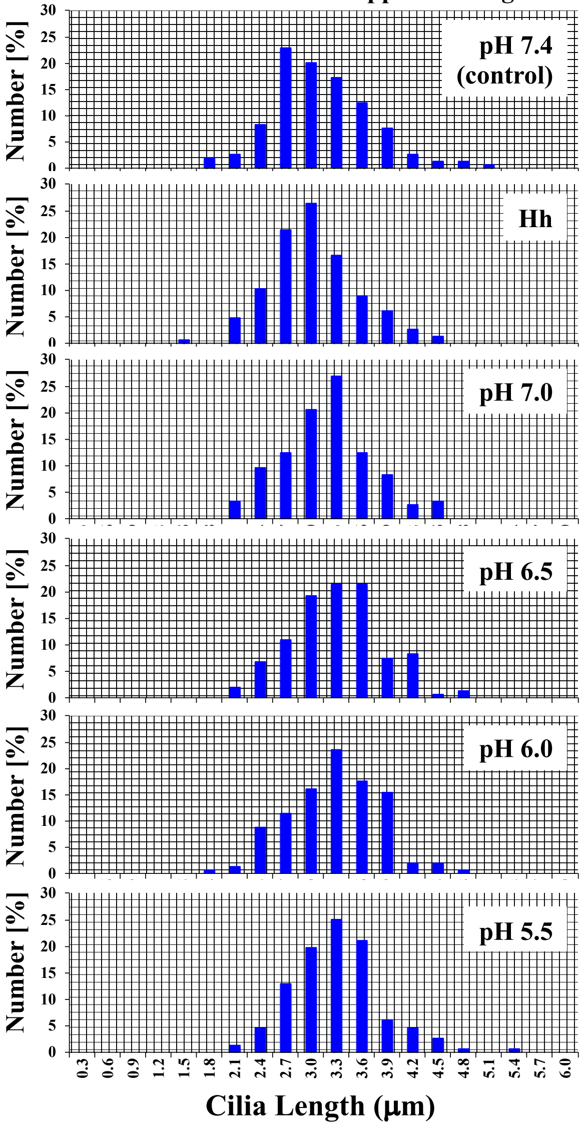

Supplement Figure 2. The lengths of primary cilia from 150 NIH3T3 cells were measured from each preparation (N=3; each 50 randomly selected cilia). These measurements are represented in the histogram to show length distribution within control, acidic pH_o or Hh activation.

Supplement Figure 3

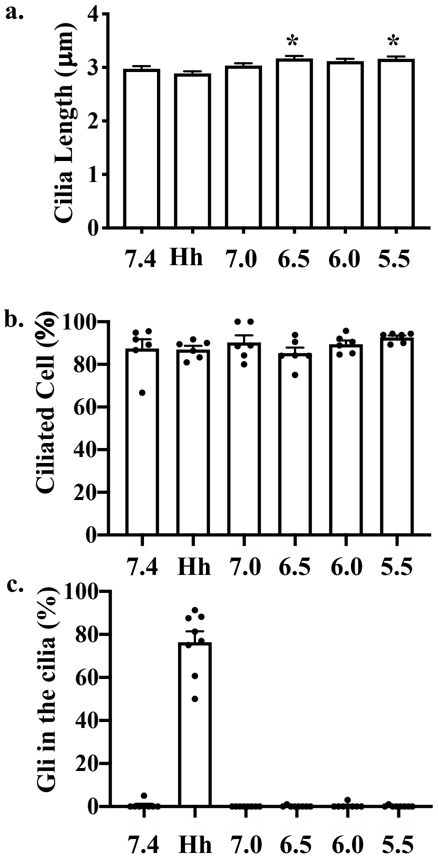

Supplement Figure 3. (a) Cilium length of NIH3T3 cells before and after Hh activation or different acidic pH_o exposures was averaged. **(b)** The percentage of cells with cilia is shown. **(c)** The percentage of cells is shown with Gli localization to the cilia. * indicates significant difference to control pH_o 7.4.

Supplement Figure 4

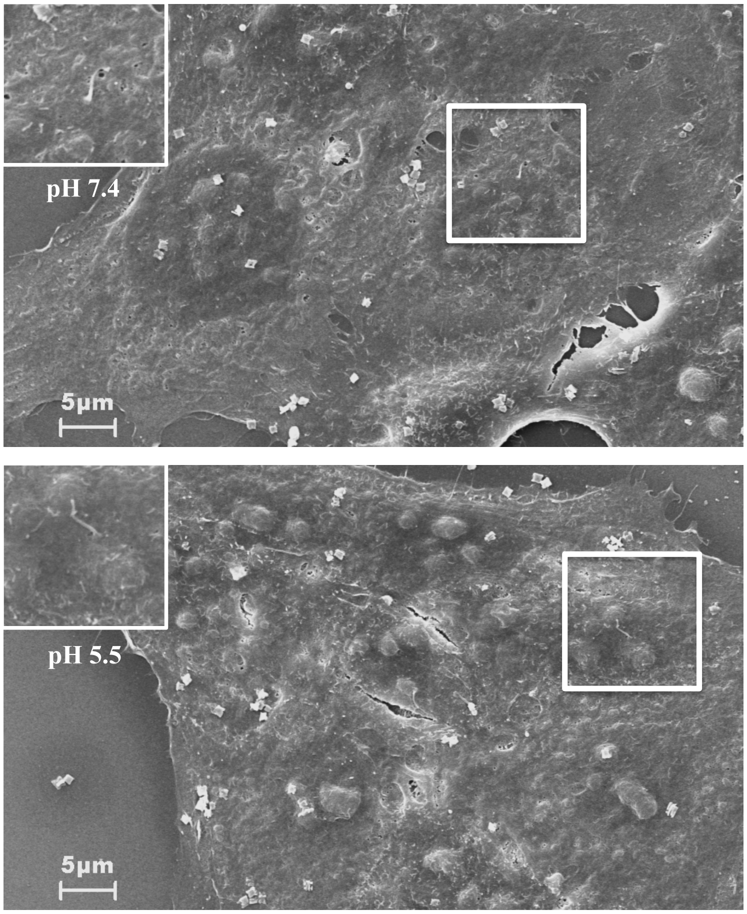

Supplement Figure 4. Electron micrographs of endothelial cells at pH 7.4 (top) and pH 5.5 (bottom). White boxes show magnified image of a single cilium.

Supplement Figure 5

a.

b.

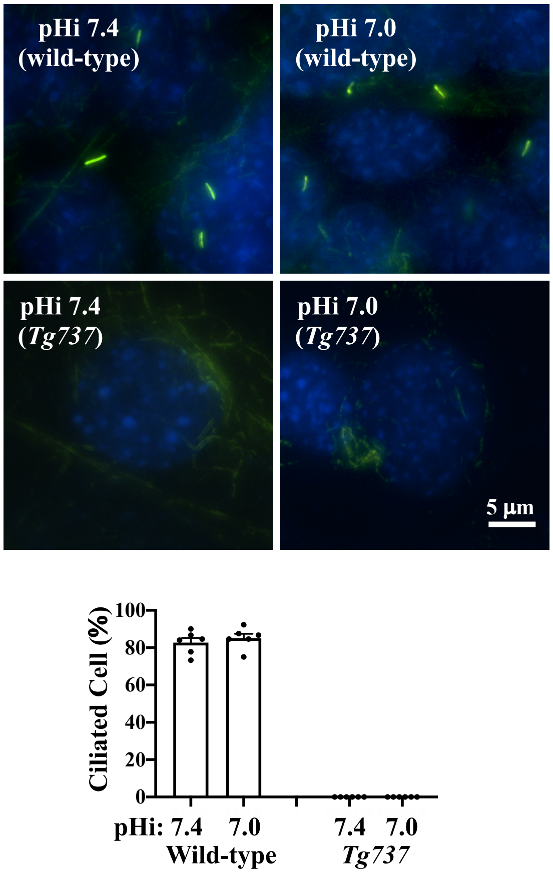

Supplement Figure 5. Endothelial cells were stained with ciliary marker (acetylated-α-tubulin; green) and nucleus marker (DAPI; blue). The lengths of primary cilia from 150 endothelial cells were measured from each preparation before and after NH_4Cl pre-pulse in 0 Na^+/K^+ solution (N=3; each 50 randomly selected cilia). **(a)** Representative images are shown for control (pH_i of 7.4) and acidic pH_i (pH_i of 7.0) in ciliated wild-type and cilia-less *Tg737* cells. **(b)** The percentage of cells with cilia is shown.

Supplement Figure 6

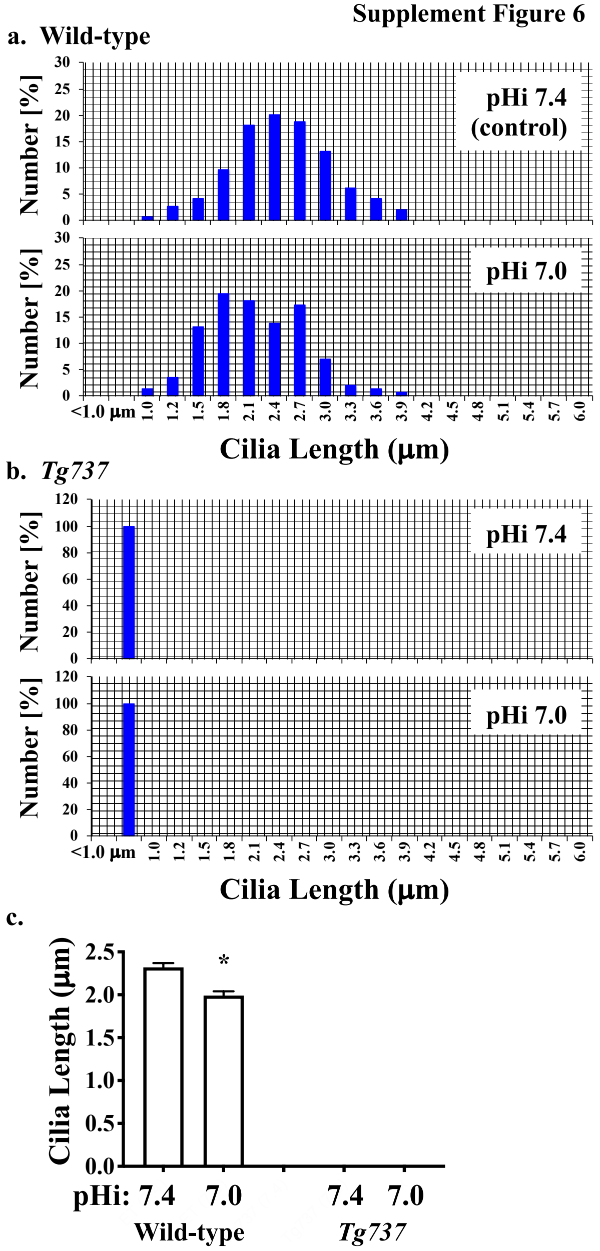

Supplement Figure 6. The lengths of primary cilia from 150 endothelial cells were measured from each preparation before and after NH_4Cl pre-pulse in 0 Na^+/K^+ solution (N=3; each 50 randomly selected cilia). These measurements are represented in the histogram to show length distribution within control (pH_i of 7.4) and acidic pH_i (pH_i of 7.0) in ciliated wild-type **(a)** and cilia-less *Tg737* **(b)** cells. **(c)** Cilium lengths before and after acidic pH_i were averaged. * indicates significant difference to control pH_o 7.4.
